# Analysis of Carbon Fiber Reinforced PEEK Hinge Mechanism Articulation Components in a Rotating Hinge Knee Design: A Comparison of In Vitro and Retrieval Findings

**DOI:** 10.1155/2016/7032830

**Published:** 2016-12-22

**Authors:** Ronja A. Schierjott, Alexander Giurea, Hans-Joachim Neuhaus, Jens Schwiesau, Andreas M. Pfaff, Sandra Utzschneider, Gianluca Tozzi, Thomas M. Grupp

**Affiliations:** ^1^School of Engineering, University of Portsmouth, Portsmouth PO1 3DJ, UK; ^2^Aesculap AG, Research & Development, Am Aesculap-Platz, 78532 Tuttlingen, Germany; ^3^Department of Orthopaedics, Vienna General Hospital, Medical University of Vienna, Waehringer Guertel 18-20, 1090 Vienna, Austria; ^4^Department of Traumatology and Orthopaedics, St. Vincenz Hospital, Am Stein 24, 58706 Menden, Germany; ^5^Department of Orthopaedic Surgery, Physical Medicine & Rehabilitation, Ludwig Maximilians University Munich, Campus Grosshadern, Marchioninistrasse 15, 81377 Munich, Germany

## Abstract

Carbon fiber reinforced poly-ether-ether-ketone (CFR-PEEK) represents a promising alternative material for bushings in total knee replacements, after early clinical failures of polyethylene in this application. The objective of the present study was to evaluate the damage modes and the extent of damage observed on CFR-PEEK hinge mechanism articulation components after in vivo service in a rotating hinge knee (RHK) system and to compare the results with corresponding components subjected to in vitro wear tests. Key question was if there were any similarities or differences between in vivo and in vitro damage characteristics. Twelve retrieved RHK systems after an average of 34.9 months in vivo underwent wear damage analysis with focus on the four integrated CFR-PEEK components and distinction between different damage modes and classification with a scoring system. The analysis included visual examination, scanning electron microscopy, and energy dispersive X-ray spectroscopy, as well as surface roughness and profile measurements. The main wear damage modes were comparable between retrieved and in vitro specimens (*n* = 3), whereby the size of affected area on the retrieved components showed a higher variation. Overall, the retrieved specimens seemed to be slightly heavier damaged which was probably attributable to the more complex loading and kinematic conditions in vivo.

## 1. Introduction

Loosening is considered among the most frequent reasons for knee arthroplasty revision [1–4], together with polyethylene (PE) debris dispersion around the implant area [5, 6].

For knee revisions and primary patients with severe varus or valgus deformities and unstable ligaments, knee arthroplasty with a rotating hinge knee (RHK) has become a viable clinical treatment [7–11]. However, traditionally applied PE hinge mechanism articulation (HMA) components/bushings may fail, mainly as a consequence of insufficient creep and wear resistance [11–13]. For this reason, different alternatives to PE have been evaluated by Grupp et al. [14, 15]. In this sense, carbon fiber reinforced poly-ether-ether-ketone (CFR-PEEK) represents an attractive alternative bearing material as it offers high creep and wear resistance and outstanding chemical resistance [16] and has already shown acceptable wear properties in hip and knee articulations [17–19].

The RHK design EnduRo® (Aesculap AG Tuttlingen, Germany) with flanges and bushings made of CFR-PEEK was clinically introduced in November 2008 and has already shown satisfying results [20]. Moreover, the biological activity in vivo of CFR-PEEK debris was reported to be comparable to PE debris [21, 22].

The suitability of CFR-PEEK as knee bearing material has been investigated in vitro on unicondylar knee arthrosplasty [18, 23]. Scholes and Unsworth [18] found that CFR-PEEK performed well and showed a lower gravimetric wear rate than conventional metal-UHMWPE (ultra-high-molecular-weight-polyethylene) articulations. However, the results of Grupp et al. [23] showed no significant wear reduction with CFR-PEEK compared to UHMWPE in low congruent fixed bearing unicompartmental knee arthroplasty (UKA) articulations and Wang et al. [24] even suggested poor performance of CFR-PEEK in high-stress nonconforming contact situations such as in tibial components of a total knee joint replacement.

Numerous retrieval studies of knee arthroplasties have been conducted with focus on the wear of the PE tibial insert [25–27]. In addition, Busanelli et al. reported a case of carbon fiber reinforced PE tibial insert which showed significantly increased wear [28]. However, to the authors' knowledge, no study has been published to date on the analysis of retrieved CFR-PEEK knee HMA components.

The main aim of this study is therefore to evaluate the wear damage modes and extent/severity of wear damage observed on retrieved CFR-PEEK HMA components in the rotating hinge knee system EnduRo in comparison with corresponding in vitro tested specimens. Hence, the key question is if there are any similarities or differences in the wear damage observed on in vivo and in vitro CFR-PEEK HMA components.

## 2. Materials and Methods

### 2.1. Implant and Clinical Data

Retrieved and in vitro tested specimens from the EnduRo rotating hinge knee system (Aesculap AG Tuttlingen, Germany) were used in this study. This mobile bearing design consisted of a multilayer coated surface with a ZrN shielding layer (Advanced Surface (AS)) or uncoated CoCr_29_Mo_6_ femoral and tibial components joint by a hinged mechanism free to rotate in flexion/extension and internal/external direction. The bearings of the hinged mechanism were made of CFR-PEEK OPTIMA LT1 CA30 (Invibio Ltd, Thornton Cleveleys, UK) consisting of 30% polyacrylonitrile (PAN) fibers and the meniscal bearing was made of UHMWPE (machined from GUR 1020), packed under nitrogen atmosphere, and sterilized using electron beam irradiation (30 ± 2 kGy) [15].

**Figure 1:**
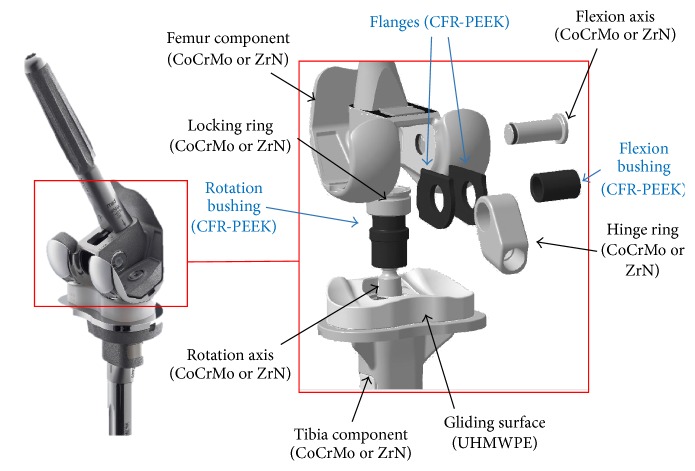
RHK EnduRo and CFR-PEEK HMA components marked (blue arrows).

The flexion bushings were articulated with the hinge ring and the flanges (outer surface) and with the flexion axis (inner surface). The rotation bushing contacted the tibial tray and the locking ring (outer surface) and the rotation axis (inner surface). The flanges were articulated with the femoral component and the hinge ring, whereby only the results for the hinge ring side are reported as there is no relative movement between the flanges and femoral component. The overall knee system including the CFR-PEEK components used in this study is shown in Figure 1. The CFR-PEEK components (Table 1) of 12 revised EnduRo knee systems in all three available implant sizes (Table 2) were analysed.

The average time in vivo (Table 2) was 34.9 ± 18.32 months for the rotation bushings and 35.6 ± 22.82 months for the flexion bushings and flanges (in case of retrieval R61 and R63, only the year of implantation was known). Aseptic loosening appeared to be a frequent reason for retrieval (6/12 cases). The patient age at implantation was known in 6/12 cases, ranging from 54 to 79 (66.7 ± 10.6) and gender distribution was 6/12 female, 3/12 male, and 3/12 unknown. For R70, no data was given.

### 2.2. Comparison with In Vitro Tests

Wear damage modes and cumulative damage scores (CDS) were compared between retrieval and CFR-PEEK components tested in vitro (*n* = 3 each) with ZrN-coated surfaces adjacent to CFR-PEEK [15]. The in vitro test with load and movement profiles according to ISO 14243-1:2002 (E) was conducted on a customized four-station (3 + 1 reference) load control servohydraulic knee-wear simulator (EndoLab® Mechanical Engineering GmbH Thansau/Rosenheim, Germany) to assess the wear behavior of the EnduRo system under simulation of level walking of an average person. The knee systems were tested over 5 Mio. cycles of 0° to 58° flexion/extension and 168 N to 2600 N axial load with a frequency of 1 Hz. Anterior-posterior (AP) motion restraint and internal-external (IE) rotation restraint were sixfold reduced (5 Nmm^−1^ and 0.1 Nmdeg^−1^) compared to ISO 14243-1:2002 (E) to simulate the absence of cruciate and collateral ligaments in the RHK treatment. Newborn calf serum (Biochrom AG Berlin, Germany) diluted with deionized water (resulting protein content 30 gL^−1^) was used as lubricant and was replaced at intervals of 0.5 Mio. cycles. Ethylene diamine tetraacetic acid and patricine were added to stabilize pH and to prevent fungal degradation. At intervals of 0.5, 1, 2, 3, 4, and 5 Mio. cycles the specimens were cleaned according to ISO 14243-2:2002 (E) and the PE gliding surface and CFR-PEEK components were analysed gravimetrically and optically.

Assuming an average of 1.76 Mio. cycles/year, 5 Mio. cycles equal approximately 2.9 years (34.8 months) in vivo [29].

The loosening torques of the retrieved rotation axis systems were compared to the resulting loosening torques after a high demanding activity in vitro test over 1 Mio. load cycles (*n* = 4), which was conducted on the EndoLab knee-wear simulator as well.

### 2.3. Loosening Torque Measurement

The loosening torques of the femoral and tibial stem, the locking ring for the rotation system, and the flexion axis were measured and compared to the nominal torques predefined for assembly in the surgery. A dial indicating torque wrench with a measurement range of ±50 Nm and minimum measurable torque of 2 Nm (Tohnichi Mfg.Co., Ltd, Tokyo, Japan) was used alongside with specific attachments for each connection (stems, locking ring, and axis). The torque wrench provided an analogue display with a memory pointer to indicate the torque required to unloose each connection.

### 2.4. Optical Wear Damage Assessment

Retrieved and in vitro CFR-PEEK components were visually examined and documented with a digital single-lens reflex (DSLR) camera (Canon EOS 650D). Scanning electron microscopy/energy-dispersive X-ray spectroscopy (SEM/EDX) analysis (Zeiss EVO 50, Oberkochen, Germany) was conducted on representative CFR-PEEK specimens.

In the visual examination, three different wear damage modes (polishing, scratching, and rims) were distinguished and quantified according to a scoring system/modified Hood-score, referring to previously conducted retrieval analysis [30–32], and reported in Table 3. In contrast to the original Hood-score, the scoring system applied within this study did not combine extent and severity but was based only on the percentage of surface area affected by the single damage modes.

A cumulative damage score (CDS) [32] per component was calculated by adding up the single scores (maximum value resulted in 3 + 3 + 3 = 9).

### 2.5. Surfaces Roughness and Profile Measurement

Surface roughness (*R*_*a*_, *R*_*z*_, and *R*_max⁡_) and surface profile were investigated according to ISO 4288 using a tactile measurement system (HOMMEL-ETAMIC TURBO WAVE V7.32, JENOPTIK AG Jena, Germany) with stylus type TKU300 and a scan length of 4.8 mm (filter according to ISO 11562).

### 2.6. Statistics

Applied statistical methods included regression (ANOVA) and Mann-Whitney tests with statistical significance set at *p* < 0.05.

## 3. Results

### 3.1. Loosening Torque Measurement

**Figure 2:**
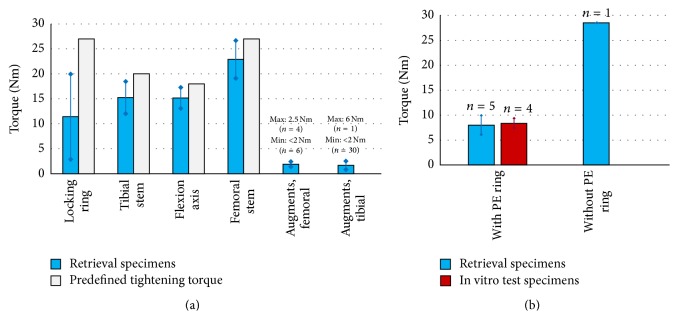
(a) Mean loosening torques in comparison with predefined tightening torque for assembly, where applicable. (b) Loosening torques of the locking ring with two different designs and comparison with in vitro test results.

The mean loosening torques (Figure 2(a)) were decreased by 57.7% (locking rings), 23.8% (tibial stem), 15.7% (flexion axis), and 15.2% (femoral stem) compared to the predefined tightening torques. In 36/41 cases the loosening torques for the augments were <2 Nm.

Five (out of six) retrieved tibial rotating systems included a locking ring with PE ring. The comparison of the loosening torques (design with PE ring: 8.0 ± 1.9 Nm, design without PE ring: 28.5 ± 0.0 Nm) showed a higher loosening torque for the new design (introduced in 2009) without PE ring.

### 3.2. Optical Wear Damage Assessment

**Figure 3:**
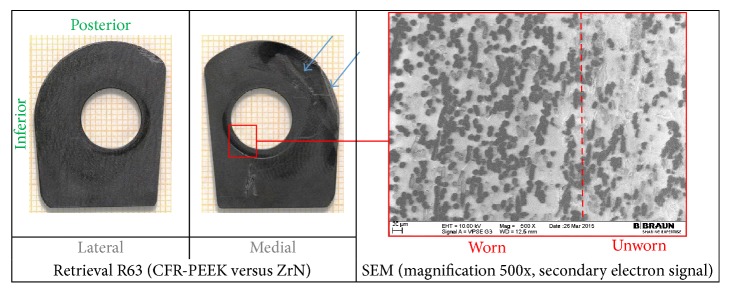
Hinge ring side of retrieved CFR-PEEK flanges with SEM image, with iatrogenic damages (blue arrows).

**Figure 4 fig4:**
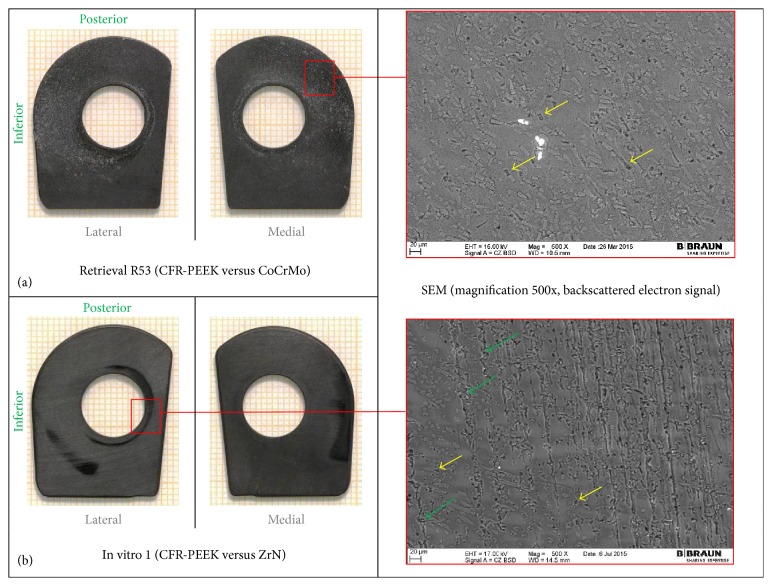
Hinge ring side of CFR-PEEK femoral flanges with SEM images. (a) Retrieved flanges adjacent to CoCrMo and (b) in vitro tested flanges adjacent to ZrN. Holes due to fiber pull-out (yellow arrows) and machining marks (green arrows) are shown.

**Figure 5 fig5:**
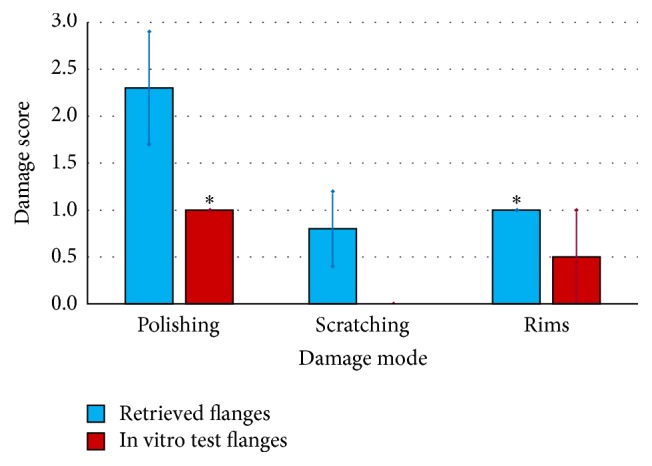
Wear damage modes on retrieved and in vitro wear tested flanges. ^*∗*^Standard deviation = 0 for equal values on all analysed specimens.

**Figure 6 fig6:**
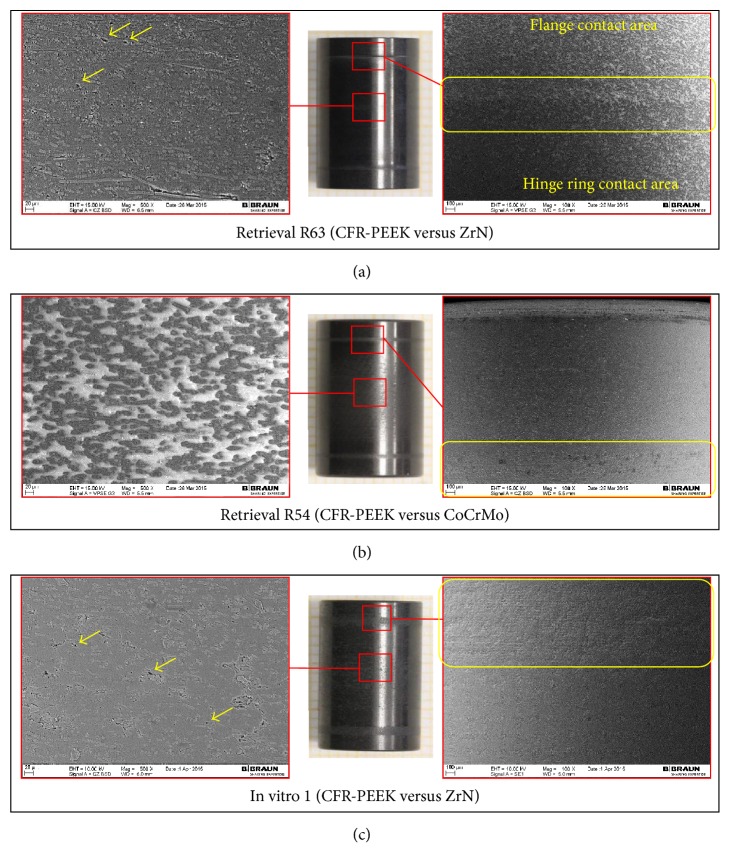
((a) and (b)) Hinge ring/flanges contact area of retrieved flexion bushings. (c) Corresponding contact area on in vitro specimen 1. Images: DSLR camera and SEM with magnification 100x (right-hand side) and 500x (left-hand side). Holes due to fiber pull-out are marked with yellow arrows.

**Figure 7 fig7:**
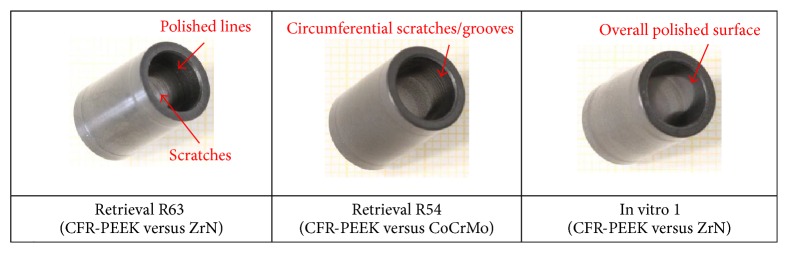
Contact surface of flexion bushings with flexion axis.

**Figure 8:**
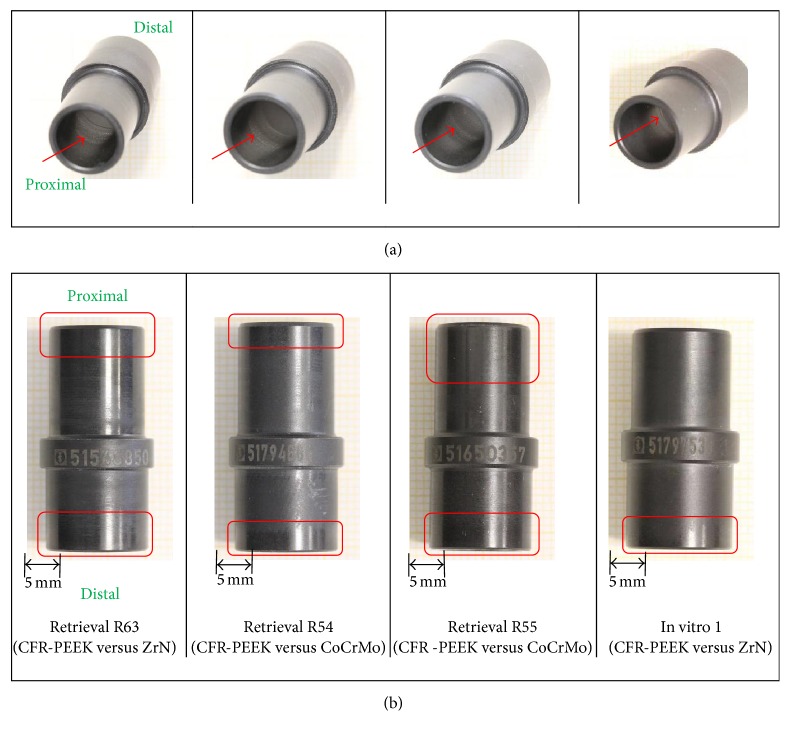
(a) Contact surface of rotation bushings with rotation axis (proximal view). (b) Contact surface of rotation bushings with tibial tray and locking ring.

The wear damage observed on retrieved and in vitro CFR-PEEK components was mainly abrasive (Figures 3 [Fig fig4] [Fig fig5] [Fig fig6] [Fig fig7]–8) with no evidence of fracture cracks or another kind of structural failure. The main wear damage mode was polishing (dark surface appearance), which was present on all retrieval and in vitro specimens.

On the flanges (Figures 3 and 4), additional rims close to the hole for the flexion bushing (all retrieved flanges and lateral in vitro flanges) and scratches in direction of movement (retrieval specimens) were observed (Figure 5). The wear damage locations on the flanges were comparable between retrieval and in vitro specimens, whereby higher variability was present on the retrieval specimens. Polishing on retrieval and in vitro specimens was mainly concentrated on the inferior-posterior part and anterior to the hole for the flexion bushing. Rims appeared superior-anterior to the hole for the flexion bushing. Some iatrogenic damages on the retrieved specimens were visible as scratches (Figure 3) or broken out edgings. In some cases, it was difficult to distinguish between iatrogenic damages and wear-induced damages, particularly between scratches and chipping.

SEM/EDX analysis was conducted on 4 retrieved flanges and one in vitro flange (selected images in Figures 3 and 4, right-hand side). All flanges showed abrasion of the PEEK matrix and exposition of roundish shaped carbon fibers in the affected areas (Figure 3, right-hand side). However, some fiber exposition was already present on the reference specimen in its initial state after manufacturing. Some more holes due to fiber pull-out were also seen on the retrieved flanges than on in vitro specimen on which machining marks were more visible (Figure 4). ZrO_2_ particles were identified with EDX on 2 retrieval specimens, which could be an indication for bone cement inclusions (Figure 4(a), right-hand side: bright particles).

Retrieved and in vitro tested flexion bushings were mainly polished on the outer surface (Figure 6) with a mean score of 2.4 ± 0.7 for retrieval and 2.0 ± 0.0 for in vitro specimens. Circumferential rims divided the hinge ring contact surface from the flanges contact surface on 7/8 retrieved specimens (mean score 0.9 ± 0.4), whereas the in vitro specimens showed circumferential lines (Figure 6, yellow boxes). SEM analysis revealed a worn out PEEK matrix in the polished regions resulting in a smooth surface appearance, as well as fragmented fibers, some holes due to fiber/fiber fragment pull-outs, and cracks along the fibers (Figure 6, left-hand side) on retrieved and in vitro flexion bushings. The number of holes seemed to be more significant for retrieval than for in vitro specimens. ZrO_2_-particles which could indicate bone cement inclusions were present on 2 out of 4 analysed retrieved flexion bushings.

The inner surfaces of the retrieved flexion bushings were, in 5/8 cases, affected by polished lines (mean score 1.6 ± 1.4) and in 4/8 cases by slight scratches (mean score 0.6 ± 0.7) in circumferential direction, whereas the corresponding surfaces of the in vitro specimens showed an overall polished appearance (Figure 7).

The outer surface of the retrieved and in vitro rotation bushings was polished (mean score 1.7 ± 0.7 for retrieval and 1.0 ± 0.0 for in vitro specimens) on the edgings (Figure 8(b), red boxes). On the inner surface of all rotating bushings, polished circumferential lines (red arrows) could be observed (mean score 1.0 ± 0.7 for retrieval and 1.3 ± 0.6 for in vitro specimens) (Figure 8(a)).

### 3.3. Cumulative Damage Score

**Figure 9:**
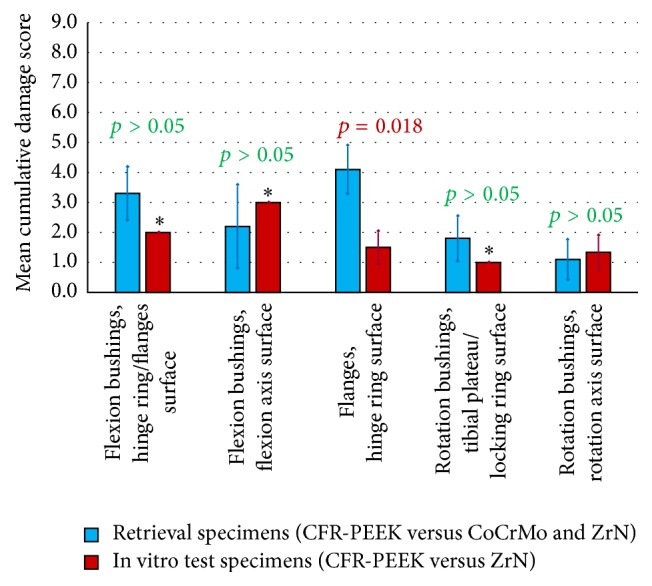
Mean cumulative damage score separated according to component type and origin. ^*∗*^Standard deviation = 0 for equal values on all analysed specimens.

Mean cumulative wear damage scores were higher for the retrieval than for the in vitro specimens, except for the axis contact surfaces of flexion and rotation bushings (Figure 9). The trend in linear regression of CDS and time in vivo was found for the hinge ring/flanges contact surface of flexion bushings (*R* ^2^ = 0.8003 and *p* = 0.0123).

### 3.4. Surfaces Roughness and Profile Measurement

**Figure 10:**
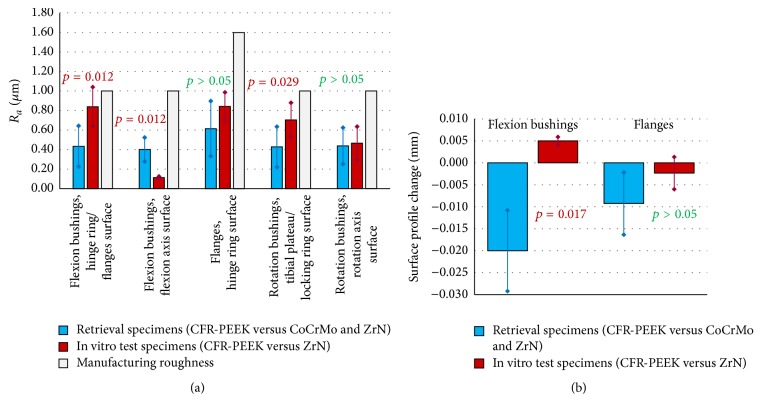
(a) Average roughness (*R*_*a*_) values of the retrieved flexion bushings, flanges, and rotation bushings in comparison with in vitro test specimens and the nominal average roughness. (b) Surface profile variations on flexion bushings and flanges.

The polishing in the worn areas was confirmed by reduced average roughness (*R*_*a*_) values in comparison with the manufacturing roughness (Figure 10(a)). The overall polished appearance on the inner surface of the in vitro flexion bushings was confirmed by the *R*_*a*_ values, more significantly decreased than for the retrieval specimens. No statistically significant correlation of decreased roughness values and time in vivo was found (*p* > 0.05 in all cases). The surface profile measurement revealed rims on 7/8 retrieved flexion bushings (hinge ring/flanges contact surface), whereas the in vitro specimens showed an elevation at the same location (Figure 10(b)). Linear regression of rim depth and time in vivo was calculated and a poor correlation was found for the flanges (*R* ^2^ = 0.4253, *p* = 0.0496).

## 4. Discussion

The main objective of the present study was to evaluate the wear damage modes and the extent of damage observed on CFR-PEEK HMA components in articulation with CoCrMo and ZrN multilayer surfaces after service in vivo in a RHK system and then compare the results with those from implant components subjected to in vitro wear testing. Key question of the study was if there existed any similarities or differences in the wear damage observed in vivo and in vitro. Retrieval and in vitro specimens showed common damage characteristics but also some differences which are both discussed in detail within the following paragraph. To the authors' knowledge, this is the first study reporting retrieval analysis of CFR-PEEK HMA components in rotating hinge type knee endoprostheses. No failure of retrieved CFR-PEEK HMA components occurred in the current study, in contrast to previously reported cases of mechanical failure of PE bushings within five months after implantation [12, 13].

### 4.1. Similarities

The observed wear damage on CFR-PEEK retrieval and in vitro specimens was mainly abrasive with polishing as main damage mode. In some cases, there was an emerging difficulty to distinguish between wear-induced and iatrogenic damages due to revision surgery, as already reported by Kurtz et al. [33]. The reduced average roughness in worn areas found in this study has been already documented for a retrieved CFR-PEEK acetabular liner adjacent to an alumina head [34] and for CFR-PEEK acetabular cups tested within a simulator study [17].

At microscopic level, the PEEK matrix was worn out and fibers were exposed, which has been previously observed on CFR-PEEK unicondylar gliding surfaces [23]. In addition, the currently analysed CFR-PEEK retrieval and the in vitro specimens showed some fragmented fibers and fiber/fiber fragment pull-outs, as well as some cracks along fibers in the worn areas that appeared smooth compared to regions in initial state.

### 4.2. Differences

Discrepancies between retrieval and in vitro specimens included circumferential rims on the retrieved flexion bushings, additional scratches in the direction of movement on the retrieved flanges, and the absence of rims on the medial in vitro flanges. The rims on the retrieved flexion bushings could be related to an increased relative movement of flexion bushings and hinge rings, maybe due to patient-related inconsistencies such as varus-valgus positioning and corresponding tilting movement, as well as muscular/soft tissue insufficiencies. Additional scratches on retrieved flanges might be due to potential bone/cement debris.

Mann-Whitney tests revealed significantly lower *R*_*a*_ values on the outer surface of the retrieved flexion bushings and significantly higher *R*_*a*_ values on their inner surface (in comparison with in vitro bushings). This indicated that the main flexion-extension movement appeared between the flexion bushing and the hinge ring (in vivo) and the bushing and the flexion axis, respectively (in vitro). This may be attributable to different activities in vivo and in vitro, as well as different movement amplitudes and frequencies, that is, varying velocity during standing up and a large number of small movements not making use of the whole range of movement available in vivo.

The cumulative wear damage scores within the present study were higher for the retrieval than for the in vitro specimens (except inner surface of rotation and flexion bushings). Harman et al. [35] also found that wear simulations underestimated the size of damage pattern and variety of damage modes in vivo for unicondylar tibial inserts. The same author reported underestimation of magnitude of damage area and extent for retrieved tibial inserts [36], a finding which is also supported by the results of Rawlinson et al. [37]. Furthermore, a higher variation of damaged areas (size of affected surface area and cumulative damage score, resp.) for retrieval than for in vitro specimens was observed. This was also found by Harman et al. who showed a considerably higher variation of damaged areas within retrieval specimens of 27 to 81% [36] and more dispersed damage pattern on retrieval than on in vitro specimens [35]. A trend for correlation of cumulative damage score and time in vivo was only found for the outer surface of flexion bushings with *R* ^2^ = 0.8003 and *p* = 0.0123, whereby the absence of a strong correlation of damage size and service time in vivo was already reported for UHMWPE unicondylar gliding surfaces [35].

ZrO_2_ particles related to bone cement fragments were documented with EDX on 4 retrieved components, but not on the in vitro specimens. This is consistent with the findings of Harman et al. [35] who observed abrasive wear with bone and/or cement on 7/17 (41%) retrieved UHMWPE unicondylar tibial inserts but not on in vitro specimens.

### 4.3. General View

It is assumed that unknown parameters such as body weight [38, 39], activity level [40], type of activity other than walking [41, 42], variations in component alignment and soft tissue restraint [43, 44], and surgical technique [45] may affect the loading conditions and hence the wear damage characteristics even more than the service time in vivo. In particular, the activity levels in patients with joint replacements were found to be highly variable [40] and greater than generally thought [46]. As in vitro knee joint wear simulations aim to reproduce the wear damage occurring in an optimally aligned, well-functioning artificial joint [36] it is reasonable that some discrepancies between retrieved and in vitro specimens appeared in the present study.

The increased loosening torque of the retrieved locking ring without PE ring (*n* = 1) underlined the efficacy of the new implant design.

A limitation of the present study may arise in the small number of samples, in particular of in vitro specimens (*n* = 3) and retrieval specimens in articulation with ZrN multilayer surface (*n* = 1), which restricted the validity of the statistical analysis. The small sample size typical of knee-wear simulator studies was previously pointed out by Harman et al. [36]. However, it is assumed that the methods applied within this preliminary study can show a statistical trend. Tactile roughness measurement was restricted to a line measurement of length 4.8 mm and depicted a spot test within the worn areas.

## 5. Conclusion

The main wear damage modes were comparable between retrieval and in vitro specimens, whereby the size of affected area on the retrieved components showed a higher variation. Overall, the retrieved specimens seemed to be slightly heavier worn, although the in vitro applied cycle number was comparable to the mean service time of the retrieval specimens. This is probably attributable to the more complex loading conditions in vivo and the fact that simulation studies aim to reproduce the wear damage which occurs in an optimally aligned knee prosthesis without taking into account patient and surgery specific variances from the ideal. Future work should include additional in vitro testing in order to enlarge the statistical power and comparisons with in vitro tests simulating high demanding activities including stair climbing, hiking, and deep squatting [29]. Wear damage related volume loss and material morphology should be assessed by means of microCT and optical interferometry for further quantification of the surface damage and to obtain information about any potential damages within the material.

## Figures and Tables

**Table 1:** Number of retrieved CFR-PEEK components.

	Flexion bushings	Rotation bushings	Medial flanges	Lateral flanges
Adjacent to CoCr_29_Mo_6_	7	11	7	7
Adjacent to ZrN	1	1	1	1

**Table 2:** Overview of clinical data, implant type, and size for retrieved CFR-PEEK rotating hinge knee components.

Specimen	Gender	Age at implantation [years]	Treated side	Implant type	Implant size (gliding surface height)	Retrieved CFR-PEEK components	Time in vivo [months]	Reason for revision
R51	F	71	Left	CoCrMo	2 (10 mm)	Rotation axis bushing	47.0	Infection/sepsis

R52	F	76	Right	CoCrMo	2 (14 mm)	Rotation axis bushing	12.0	Infection/sepsis
						Flexion axis bushing	41.0	
						Femoral flanges	41.0	

R53	F	79	Left	CoCrMo	2 (20 mm)	Rotation axis bushing	54.0	Aseptic loosening
						Flexion axis bushing	54.0	
						Femoral flanges	54.0	

R54	F	Unknown	Left	CoCrMo	2 (12 mm)	Rotation axis bushing	12.0	Poor mobility, postoperative
						Flexion axis bushing	12.0	
						Femoral flanges	12.0	

R55	M	55	Left	CoCrMo	3 (10 mm)	Rotation axis bushing	43.0	Infection/sepsis

R60	F	54	Left	CoCrMo	2 (14 mm)	Rotation axis bushing	40.5	Aseptic loosening
						Flexion axis bushing	40.5	
						Femoral flanges	40.5	

R61	M	Unknown	Unknown	CoCrMo	3 (12 mm)	Rotation axis bushing	55.0	Aseptic loosening and bone perforation with stem

R63	F	Unknown	Left	ZrN	2, customized (12 mm)	Rotation axis bushing	60.0	Aseptic loosening
						Flexion axis bushing	60.0	
						Femoral flanges	60.0	

R67	M	65	Left	CoCrMo	3 (10 mm)	Rotation axis bushing	20.0	Aseptic loosening

R70	Unknown	Unknown	Left	CoCrMo	2 (10 mm)	Rotation axis bushing	Unknown	Unknown
						Flexion axis bushing	Unknown	
						Femoral flanges	Unknown	

R71	Unknown	Unknown	Right	CoCrMo	1 (10 mm)	Rotation axis bushing	22.0	Aseptic loosening
						Flexion axis bushing	22.0	
						Femoral flanges	22.0	

R72	Unknown	Unknown	Right	CoCrMo	3 (10 mm)	Rotation axis bushing	18.0	Infection/sepsis
						Flexion axis bushing	20.0	
						Femoral flanges	20.0	

**Table 3:** Scoring system for optical wear damage assessment.

Score	Damage description
0	Surface not affected by damage type
1	Extent of surface affected: 0 < *x* < 30%
2	Extent of surface affected: 30 ≤ *x* < 60%
3	Extent of surface affected: *x* ≥ 60%
